# Identification of Primary and Metastatic Lung Cancer-Related lncRNAs and Potential Targeted Drugs Based on ceRNA Network

**DOI:** 10.3389/fonc.2020.628930

**Published:** 2021-02-03

**Authors:** Siyao Dong, Cheng Wu, Chengyan Song, Baocui Qi, Lu Liu, Yan Xu

**Affiliations:** College of Bioinformatics Science and Technology, Harbin Medical University, Harbin, China

**Keywords:** ceRNA network, lncRNA, lung cancer, primary cancer, metastatic cancer

## Abstract

Lung cancer metastasis is the leading cause of poor prognosis and death for patients. Long noncoding RNAs (lncRNAs) have been validated the close correlation with lung cancer metastasis, but few comprehensive analyses have reported the specific association between lncRNA and cancer metastasis, especially *via* both competing endogenous RNA (ceRNA) regulatory relationships and functional regulatory networks. Here, we constructed primary and metastatic ceRNA networks, identified 12 and 3 candidate lncRNAs for lung adenocarcinoma (LUAD) and lung squamous cell carcinoma (LUSC) respectively and excavated some drugs that might have potential therapeutic effects on lung cancer progression. In summary, this study systematically analyzed the competitive relationships and regulatory mechanism of the repeatedly dysregulated lncRNAs in lung cancer carcinogenesis and metastasis, and provided a new idea for screening potential therapeutic drugs for lung cancer.

## Introduction

Lung cancer is the leading cause of cancer-related mortality worldwide (1), the five-year survival rate for patients diagnosed with advanced-stage lung metastasis is only 5% (2). Therefore, lung cancer metastasis is closely related to the poor prognosis of lung cancer patients. Studies have shown that lung cancer invasion and metastasis are due to the accumulation of changes in various gene expressions, structural features and functions (3). Invasion and metastasis of lung cancer is also a multi-step, multi-factor complex process involving loss of adhesion between tumor cells, degradation of tumor extracellular matrix, enhancement of tumor cell migration, tumor neovascularization, and other cellular biological behaviors. And these specific biological behaviors are more regulated by specific genes (4). However, the current mechanism of lung cancer metastasis is still insufficiently understood.

LncRNAs involve in lung cancer carcinogenesis and metastasis directly (5–7). For example, MALAT1 was significantly highly expressed in non-small cell lung cancer(NSCLC) with bone metastasis and in NSCLC cell lines with high bone metastatic ability (8); An lncRNA, actin filament associated protein 1 antisense RNA1 (AFAP1-AS1), was the most significantly upregulated in lung cancer and associated with poor prognosis (9). Nowadays, the study that systematically identifies lncRNAs and their mechanisms of action associated with the development and metastasis of lung cancer is still rare.

In recent years, more and more studies have confirmed that lncRNAs can interact with microRNAs (miRNAs) as competitive endogenous RNA, and regulate cancer progression and metastasis by involving in the regulation of target gene expression. LncRNA *RSF1-IT2* was found to function as ceRNA, sponging *miR-129-5p*, which targets *SNAI1*. Components of the *HMGB1-RSF1-IT2-miR-129-5p-SNAI1* pathway may have a potential as prognostic and therapeutic targets in lung cancer (10). Thus, by identifying the competitive relationships in which lncRNAs are involved, the function of lncRNAs can be revealed and possible intervention targets are provided. On the other hand, the research and development of new drugs requires a lot of manpower and material resources, and the process is very complicated with high risks and low success rates. Developing the potential efficacy of known drugs can reduce the cost of new drug development and shorten the time of drug development. Lately, some researchers have validated that non-coding RNA can be used as a drug target for disease treatment (11).

LUAD and LUSC are the two most main cancer types of lung cancer, accounting for 50 and 30% of lung cancer, respectively, and belong to non-small cell lung cancer (NSCLC). However, there is a difference in the risk of cancer metastasis between them, and this risk of metastasis is more regulated by different genes. This study analyzed normal, primary, and metastatic lung cancer (LUAD, LUSC) samples from The Cancer Genome Atlas (TCGA) database, identified repeatedly dysregulated lncRNAs during carcinogenesis and cancer metastasis, and explored related functional changes in the process of lung cancer carcinogenesis and metastasis by constructing lncRNA-related primary and metastatic gain/loss ceRNA networks. Further we identified candidate lncRNAs and their associated competing triplets in lung cancer (LUAD, LUSC) carcinogenesis and metastasis, discovered some potential targeted drugs for the treatment of lung cancer carcinogenesis and metastasis by drug resetting, and provided a new class of molecular markers for lung cancer prediction and diagnosis.

## Materials and Methods

### Data Collection

For lung cancer, mRNA expression profiles were downloaded from TCGA data portal (12). LncRNA expression profiles were obtained from The Atlas of Noncoding RNAs in Cancer (TANRIC) (13). LncRNAs and mRNAs express in at least 70% samples were retained. We normalized expression values of lncRNA and mRNA by logarithmic transforming. Besides, clinical data of samples were also downloaded from TCGA which provided Ajcc staging information of lung cancer, including Ajcc tumor pathologic pt(T), Ajcc nodes pathologic pn(N), Ajcc metastasis pathologic pm(M), and Ajcc pathologic tumor stage(Stage). T, N, and M represent the size of the primary tumor, the status of regional lymph nodes metastasis and distant metastasis, respectively.

### Differential Expression Analysis

Differentially expressed lncRNAs were identified *via* t-test and fold change (FC), based on lncRNA expression in normal, primary and metastatic samples. Here, lncRNA with a threshold of p <0.05, |FC| >1.2 was considered up-regulated and |FC| <1/1.2 was considered down-regulated.

### Collection of miRNA-Target Interactions

MiRNA-target interactions (miRNA-lncRNA, miRNA-mRNA) were downloaded from a database, Starbasev2.0 (14). Furthermore, experimentally validated non-weak miRNA-mRNA interactions were derived from mirTarBase (15), human mature miRNA names were retrieved from MirBase (16), and then high-throughput HITS-CLIP and PAR-CLIP experimental miRNA-lncRNA interactions were obtained from LncBase v2.0 (17).

### Identification of Competitive Pairs

Aiming at identifying potential competitive lncRNA-mRNA pairs, we evaluated the significance of the shared miRNAs between each pair by means of the hypergeometric test. Given an lncRNA A, mRNA B, their enrichment significance was calculated according to the formula:

$$\text{P}\;=\;1-\sum_{\text{t}=0}^{\text{x}}\frac{\left({}_{\text{t}}^{K}\right)\left({}_{\text{M}-\text{t}}^{\text{N}-\text{K}}\right)}{\left({}_{\text{M}}^{N}\right)}$$

Where N was the number of all target miRNAs, K and M were the number of miRNAs associated with the current A and B, and x was the number of common miRNAs shared by A and B. The significant p values were subjected to false discovery rate (FDR) correction. The pairs with FDR value less than 0.05 were considered as ceRNA pairs. Positive Pearson correlation coefficient (PCC) ranked in the top 10% with a p-value threshold of 0.05 was used to determine co-expression relationships between lncRNA and mRNA (Benjamini-Hochberg, FDR < 0.05). Briefly, lncRNA-mRNA pairs which simultaneously meet the standard of the hypergeometric test and co-expression relationship were defined as competitive pairs.

### Construction of the Gain/Loss ceRNA Networks for Primary/Metastatic Lung Cancer

For competitive pairs in primary LUAD samples, the same competitive pairs in normal samples were removed and specific competitive pairs were retained to construct the primary gain ceRNA network of LUAD. After deleting the same competitive pairs in primary LUAD samples, special competitive pairs in normal samples were used to construct the primary loss ceRNA network of LUAD. Similarly, after comparing the competitive pairs between primary and metastatic LUAD samples, we constructed the metastatic gain ceRNA network of LUAD and the metastatic loss network of LUAD. Meanwhile, the process on LUSC was the same as LUAD, which means that a total of 8 networks were constructed for lung cancer. These networks were visualized by CytoScape (18) (http://cytoscape.github.io/). Gene Ontology (GO) (19) functional annotation and Kyoto Encyclopedia of Genes and Genomes (KEGG) (20) pathway analysis were executed on DAVID bioinformatics resources (21) to determine the principal functions of the networks. Functional categories with FDR <0.01 were considered statistically significant in our analyses.

### Survival Analysis

To estimate whether candidate lncRNAs can divide patients into high-risk and low-risk groups significantly, we extracted clinical information of samples and carried out univariate survival analysis for each lncRNA. Survival analysis was conducted by R package “survival” (22). Kaplan-Meier survival curves were utilized to assess overall survival time of patients. Log-rank tests were performed to evaluate the survival differences between the two curves.

## Results

### Classification of Lung Cancer Samples

Primary/metastatic samples were classified according to the clinical information. After analyzing TNM (Tumor Node Metastasis) and stage grouping classification information, we determined primary samples (**Supplementary Table 1**). Metastatic samples had lymph metastasis (N1,2) or distant metastasis (M1). Therefore, samples at Stage2 (N1, 2) or Stage3/Stage4 (T4/N1,2,3 M1) were considered as metastatic samples (**Supplementary Table 1**).

**Figure 1:**
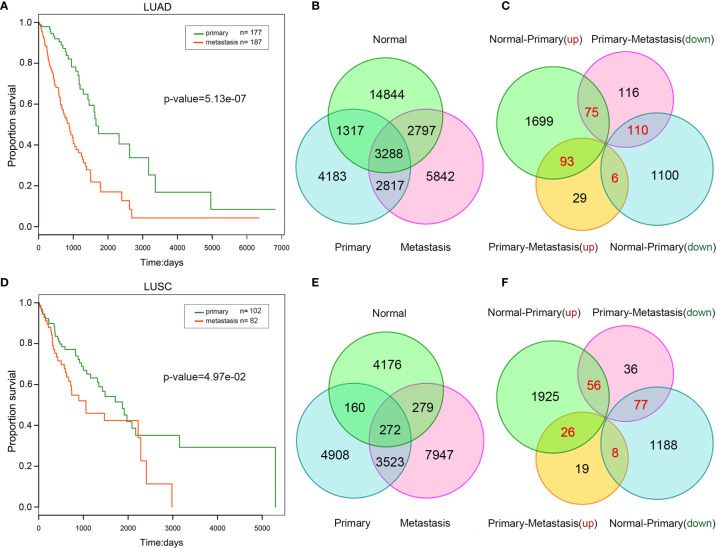
Comparison between primary and metastatic lung cancer. Kaplan–Meier survival curve analysis for overall survival of LUAD **(A)** /LUSC **(B)**. The number of competitive pairs in normal, primary, and metastatic LUAD **(C)** /LUSC **(D)**. The number of up/down-regulated lncRNAs (normal VS. primary, primary VS. metastatic) in LUAD **(E)** /LUSC **(F)**. The number of repeatedly dysregulated lncRNAs is labeled by red color.

Because metastatic lung cancer samples usually have poorer prognosis, we compared the survival time between primary and metastatic samples. Metastatic samples tended to present higher death ratio and shorter survival time in lung cancer (**Supplementary Table 2**). Additionally, Univariate Cox regression analysis revealed a significant association between patient survival time and metastasis in lung cancer (**Figures 1A, B**). Our findings demonstrated the reliability of the primary and metastatic samples we used and the importance of studying lung cancer metastasis.

### Construction of Lung Cancer ceRNA Networks

To construct ceRNA network, we first predicted potential lncRNA-mRNA competitive pairs by means of the hypergeometric test on the basis of miRNA-lncRNA and miRNA-mRNA interactions. Secondly, the PCCs of the pairs in each status (normal/primary/metastasis) were calculated respectively to filter out lncRNA-mRNA pairs with a significant positive PCC ranked in the top 10%. Finally, lncRNA-mRNA pairs with a significant positive PCC and satisfied the significance of the hypergeometric test was referred to mutually competitive pairs (**Figures 1C, D**, and **Supplementary Table 3**).

In our perspective, changes among normal-primary-metastatic progress probably are due to the changes of some competitive relationships that some key lncRNAs involved in. More importantly, it is well known that differentially expressed lncRNAs are more likely to play key roles in the progression of carcinogenesis and cancer metastasis (23). Next, we identified differentially up/down-regulated lncRNAs in lung cancer (**Supplementary Table 4**) between different statuses (normal vs. primary, primary vs. metastasis), respectively. Thus, four types of lncRNAs were mined: lncRNAs were up-regulated from normal to primary status, and continuously up-regulated (up-up) or reversely down-regulated (up-down) from primary to metastatic cancer; lncRNAs were down-regulated from normal to primary, and continuously down-regulated (down-down) or reversely up-regulated (down-up) from primary to metastatic cancer (**Figures 1E, F**). These repeatedly dysregulated lncRNAs in cancer progression mentioned above may play a key role in carcinogenesis and influence cancer metastasis.

Moreover, we investigated competitive pairs that comprised of repeatedly dysregulated lncRNAs to find out it is “gain” or “loss” leads to carcinogenesis and cancer metastasis in cancer progression. Then, repeatedly dysregulated lncRNAs were mapped back to the corresponding gain/loss competitive pairs, and the gain/loss ceRNA networks were constructed (**Supplementary Table 5**), respectively.

### LncRNAs Affect Cancer Functions by Competing for mRNAs

Based on mRNAs in the ceRNA network, we performed GO function and KEGG pathway enrichment analysis to explore significant biological processes and correlated pathways in the primary and metastatic gain/loss ceRNA networks.

**Figure 2:**
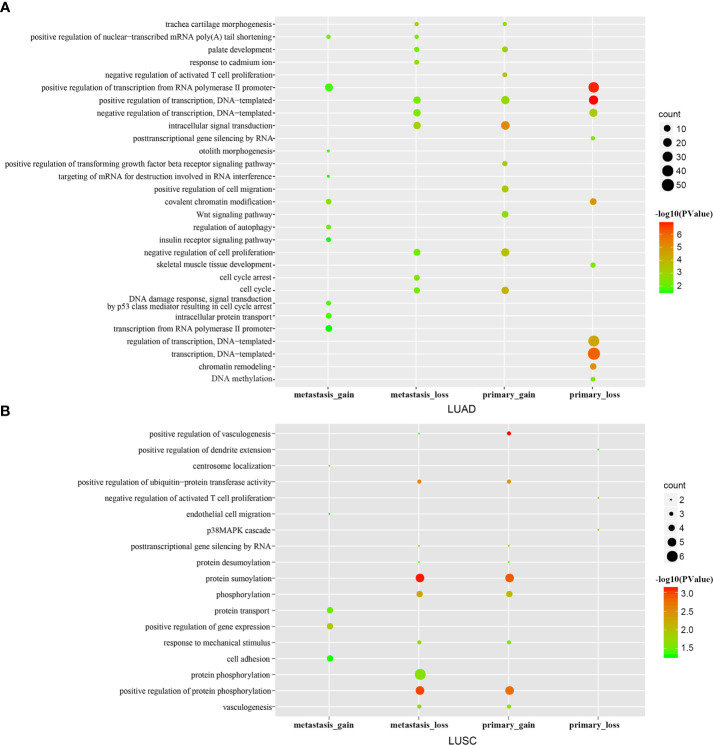
GO enrichment analysis. The top 10 significantly enriched GO terms of the primary/metastatic gain/loss networks in LUAD **(A)** /LUSC **(B)**.

For LUAD, the results revealed that genes in the primary gain ceRNA network were enriched for multiple categories related to cell proliferation such as intracellular signal transduction, cell cycle, cell proliferation, T cell proliferation, and cell migration (**Figure 2A**); However, genes in the primary loss ceRNA network were mainly enriched in processes about normal life activities, including transcription, DNA-templated, and chromatin remodeling. Comparison of the functions of the gain and loss ceRNA networks of metastatic LUAD implicated that genes in the metastatic gain ceRNA network were mainly enriched in processes regarding cancer metastasis. On the other hand, genes in the metastatic loss ceRNA network were mainly enriched in processes about normal life activities and cancer, for example positive regulation of transcription, DNA-templated, cell cycle and cell cycle arrest. The same process was carried out for LUSC (**Figure 2B**). These results suggested that lung cancer carcinogenesis, resulting from losing some normal functions of cells as well as gaining some cancer-related functions.

**Figure 3:**
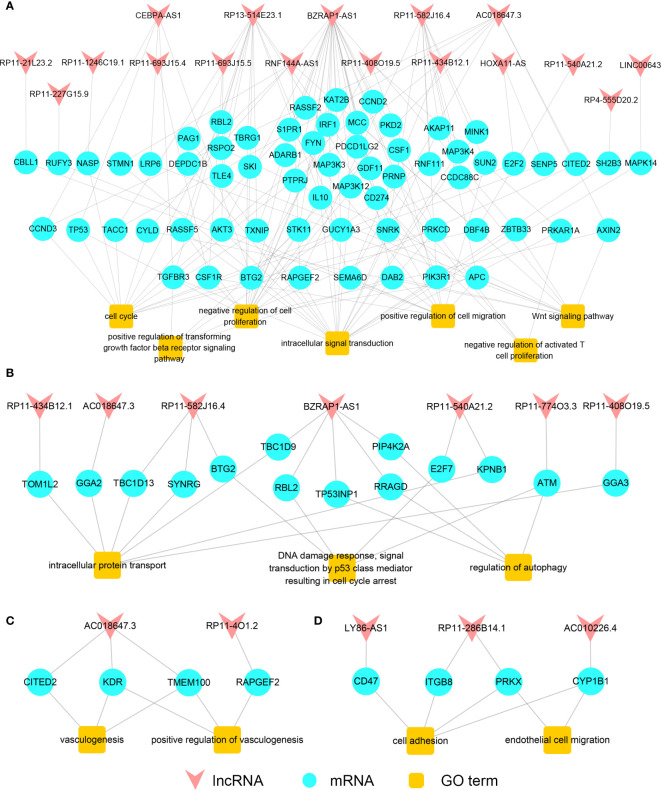
The functional regulatory networks. The primary **(A)** /metastatic **(B)** functional regulatory gain network of LUAD. The primary **(C)** /metastatic **(D)** functional regulatory gain network of LUSC.

In order to explore the association between lncRNA and cancer-related functions gained from the process of carcinogenesis and cancer metastasis, we built the primary functional regulatory gain network of LUAD (**Figure 3A**) using seven carcinogenesis-related functions of the primary gain network. Notably, most cancer-related functions were found regulated by a few lncRNAs. For example, “negative regulation of cell proliferation” that regulated by 16 mRNAs was one of critical functions in carcinogenesis, and eight mRNAs among them were regulated by lncRNA BZRAP1-AS1, indicating that BZRAP1-AS1 played an important role in cell proliferation. “Intracellular signal transduction” regulated by 20 mRNAs, and 13 mRNAs among them were regulated by lncRNA BZRAP1-AS1, RP13-514E23.1, and RP11-582J16.4. Thus, lncRNAs that had a higher degree and regulated more cancer-related functions may be more likely associated with carcinogenesis. Genes in the metastatic gain network of LUAD enriched in many processes, including intracellular protein transport, regulation of autophagy and DNA damage response and so on. It has been reported previously that if cancer cells metastasize, they must be detached from original tissues firstly and bind with proteins (24), while “intracellular protein transport” contributes to gaining specific proteins for cancer cells; there are also some studies documented that “regulation of autophagy”, “DNA damage response”, and “signal transduction by p53 class mediator resulting in cell cycle arrest” play significant roles in cancer metastasis (25). Thus, we built the metastatic functional regulatory network of LUAD by the use of the three cancer-related functions (**Figure 3B**) mentioned above. LncRNA *BZRAP1-AS1* was found not only was it crucial in the primary functional regulatory network, but also it was the node with the maximum degree in the metastatic functional regulatory gain network, and was critical for cancer metastasis, influencing the three functions by means of interacting with *PIP4K2A*, *TP53INP1*, *RRAGD*, *TBC1D9*, and *RBL2*. Furthermore, we found six among seven lncRNAs that regulating metastasis-related functions in LUAD also regulated cancer-related functions in the primary functional regulatory network. Some lncRNAs may play different roles in different cancer statuses by competing with different mRNAs to regulate LUAD carcinogenesis and metastasis.

Likewise, for LUSC, we built the primary functional regulatory gain network by the functions “positive regulation of vasculogenesis” and “vasculogenesis”. According to the network, vasculogenesis was mainly regulated by lncRNA *AC018647.3* in the process of LUSC carcinogenesis (**Figure 3C**). Then, we built the metastatic functional regulatory gain network using functions related to cancer metastasis. The cancer metastasis-related functions “cell adhesion” and “endothelial cell” were found regulated by lncRNA *LY86-AS1*, *AC010226.4*, *RP11-286B14.1* mainly (**Figure 3D**). It comprehensively demonstrated that the pivotal roles that lncRNAs played in the functional networks during the process of lung cancer carcinogenesis and metastasis could be an important indicator for identifying lung cancer candidate biomarkers.

### Identification of Candidate lncRNAs

**Figure 4:**
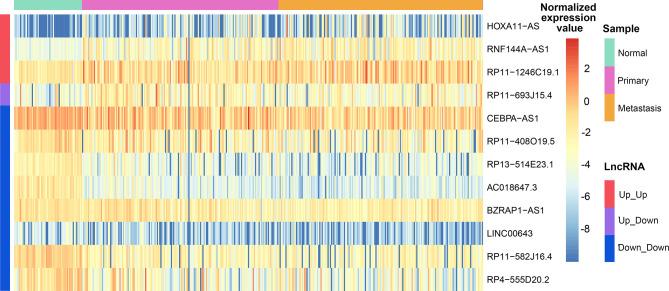
The expression heatmap of 12 candidate lncRNAs in LUAD.

Closeness is an important feature for network, and hub node is a pivotal node with an extremely high level of closeness. Nodes with degree >=5 in the gain/loss ceRNA network were defined as hub, and then we detected hubs in the ceRNA networks of LUAD. Fourteen lncRNAs among all hubs were not only associated with LUAD carcinogenesis, but also correlated with cancer metastasis. Twelve lncRNAs among the 14 were found in the primary and metastatic functional regulatory gain networks, providing that the 12 lncRNAs were important in both ceRNA networks and functional regulatory networks and could be used as candidate biomarkers for LUAD carcinogenesis and metastasis. Three lncRNAs among the 12, *HOXA11-AS*, *RNF144A-AS1*, and *RP11-1246C19.1*, were continuously up-regulated among normal-primary-metastatic progress; lncRNA *RP11-693J15.4* was up-regulated between normal-primary while reversely down-regulated between primary-metastatic cancer, and the other eight lncRNAs were continuously down-regulated among normal-primary-metastatic progress (**Figure 4**). We found that *HOXA11-AS* was continuously up-regulated in LUAD carcinogenesis as well as metastasis and regulated the expression of *E2F2* and *SENP5* by way of competing for miRNAs in the primary functional regulatory network to influence cell cycle, implying that *HOXA11-AS* was closely related to lung cancer carcinogenesis.

**Figure 5:**
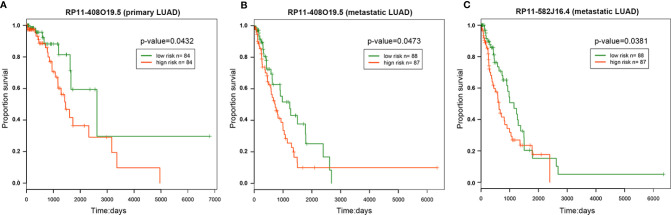
Survival analysis. The survival analysis of RP11-408O19.5 for primary **(A)** and metastatic **(B)** LUAD. **(C)** The survival analysis of RP11-582J16.4 for metastatic LUAD.

The results of univariate survival analysis showed that lncRNA *RP11-408O19.5* among 12 candidate lncRNAs could classify LUAD samples into high and low risk groups significantly. It was not only related to the survival time of primary samples (**Figure 5A**), but also affected the survival of metastatic samples (**Figure 5B**); LncRNA *RP11-582J16.4* influenced the survival of metastatic samples (**Figure 5C**).

For LUSC, six lncRNAs as hub nodes were identified not only in the primary network, but also in the metastatic network. *AC018647.3*, *RP11-4O1.2*, and *RP11-286B14.1* among the six lncRNAs were found in the functional regulatory network of LUSC, suggesting the important roles of the three lncRNAs in LUSC metastasis.

### Prediction of Potential Targeted Drugs for Lung Cancer

**Figure 6:**
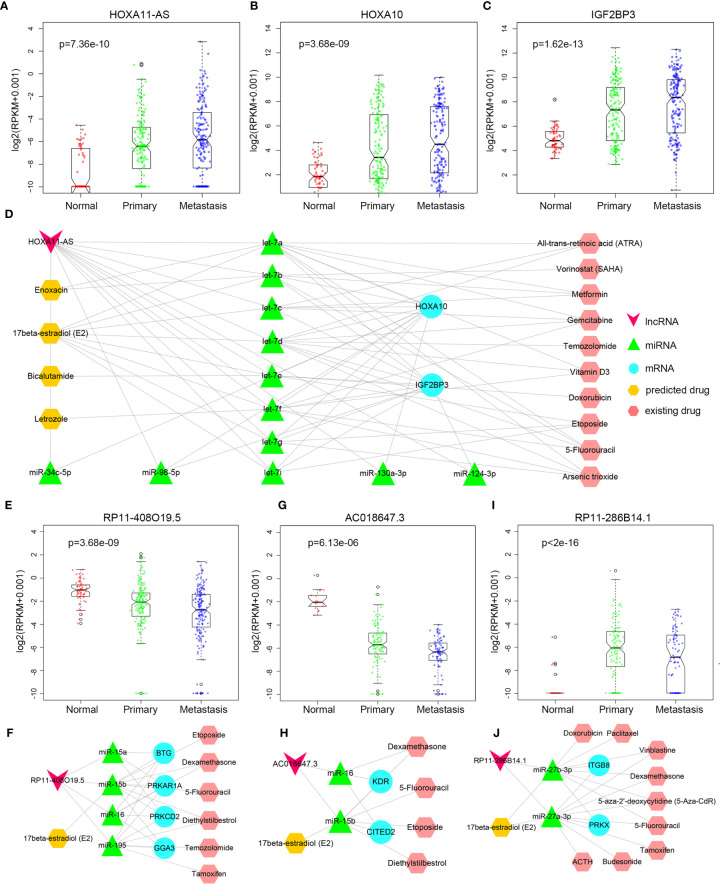
Drug prediction. **(A–C)** The expression difference of HOXA11-AS, HOXA10, and IGF2BP3 between normal, primary, and metastatic LUAD. **(D)** HOXA11-AS related drug regulation network. **(E)** The expression difference of RP11-408O19.5 between normal, primary, and metastatic LUAD. **(F)** RP11-408O19.5 related drug prediction in LUAD. **(G)** The expression difference of AC018647.3 between normal, primary, and metastatic LUSC. **(H)** AC018647.3 related drug prediction in LUSC. **(I)** The expression difference of RP11-286B14.1 between normal, primary, and metastatic LUSC. **(J)** RP11-286B14.1 related drug prediction in LUSC.

In recent years, new directions for RNA-targeted drug research have received more and more attention. Some studies have reported the association of *HOXA11-AS* and NSCLC (26–28). *HOXA11-AS* was continuously up-regulated in primary and metastatic LUAD (**Figure 6A**). *HOXA11-AS* gained some competitive relationships in primary cancer, and part of competitive relationships were remained to metastatic cancer, indicating that *HOXA11-AS* might play a significant role in the progression of LUAD. Accordingly, for *HOXA11-AS*, as long as we could figure out some drugs that inhibit its upregulation, it was believed that primary and metastatic LUAD would be inhibited. In primary or metastatic cancer, mRNAs *IGF2BP3*, *HOXA9*, *HOXA10*, *CEBPG* competed with *HOXA11-AS* mutually, and *HOXA10* (**Figure 6B**) as well as *IGF2BP3* (**Figure 6C**) were also continuously up-regulated among normal-primary-metastatic process with significant difference in variance analysis. Currently, some studies have demonstrated the association among *HOXA10*, *IGF2BP3*, and lung cancer (29, 30). The results further validated the importance of competitive relationships among *HOXA11-AS*, *IGF2BP3*, and *HOXA10* in lung cancer progression. Besides, *HOXA11-AS*, *IGF2BP3*, and *HOXA10* regulated mutually by competing for let-7 family (**Figure 6D**). The let-7 family has been reported the close association with lung cancer (31). No drug was found targeting at the 3 lncRNAs. However, designing anti-cancer drugs targeting miRNAs can regulate the expression of related genes at the initial stage of transcription, reduce energy and resources, and exert pharmacological effects more effectively. Thus drug information targeting miRNAs was downloaded from SM2miR (32), and 14 FDA-approved drugs upregulating expression of let-7 family were screened out. Ten drugs among them have been proved to have an effect on lung cancer, such as Etoposide and Gemcitabine have been widely used in LUAD clinical treatment (33, 34). Among the other 4 drugs, Bicalutamide is commonly used to treat prostate cancer (35); Letrozole is used to treat breast cancer (36); Enoxacin has a strong bactericidal effect (37); 17beta-estradiol (E2) promotes development of reproductive system and maintains reproductive function, with protective effects, anti-inflammatory, anti-oxidative, and anti-apoptotic effects (38). The four drugs were considered could be candidate drugs for treating lung adenocarcinoma probably.

LncRNA *RP11-408O19.5* was associated with the survival of primary and metastatic LUAD patients, so it was important for *RP11-408O19.5* to be predicted related drugs to increase survival time of patients. It was continuously down-regulated in primary and metastatic LUAD (**Figure 6E**). MRNAs *PRKCD*, *PRKAR1A*, *BTG2* competed with lncRNA *RP11-408O19.5* for the same miRNAs (*miR-15a-5p*, *miR-15b-5p*, *miR-16-5p*, *miR-195-5p*) in primary cancer; lncRNA *RP11-408O19.5* competed with *GGA3* for *miR-15b-5p*, *miR-16-5p*, and *miR-195-5p* in metastatic cancer (**Figure 6F**). Interestingly, even though the competitive mRNA changed, the miRNA as a bridge was basically the same. If we only focused on the same miRNAs (*miR-15b-5p*, *miR-16-5p*, and *miR-195-5p*) in primary and metastatic cancer, and regulated expression of the miRNAs, the drug which has an effect on primary and metastatic LUAD would be predicted. That is, cancer metastasis would be inhibited in the process of inhibiting carcinogenesis. From SM2miR, the drugs Diethylstilbestrol, Etoposide, 5-Fluorouracil, 17beta-estradiol (E2), Dexamethasone, Temozolomide and Tamoxifen which could downregulate the three mRNAs (*PRKCD*, *PRKAR1A*, *BTG2*) were identified. Six drugs excluding 17beta-estradiol (E2) were validated concerning treatment for lung cancer in some studies (33, 39–42) yet 17beta-estradiol (E2) was also a targeted drug predicted by lncRNA *HOXA11-AS*, further indicating that 17beta-estradiol (E2) could be a candidate drug in the process of LUAD metastasis.

Similarly, we also predicted targeted drugs for LUSC. LncRNA *AC018647.3* was continuously up-regulated during the primary and metastatic processes (**Figure 6G**). It had competitive relationships with four mRNAs (*KDR*, *CITED2*, *TMEM100*, and *RAPGEF2*, **Figure 6H**), *KDR* and *CITED2* among them were reported the association with NSCLC (43, 44). Then the miRNAs (*miR-15b-5p* and *miR-16-5p*) that the lncRNA *AC018647.3* competed with *KDR* and *CITED2* in primary of LUSC were identified. Finally, five drugs (17beta-estradiol (E2), Diethylstilbestrol, Etoposide, 5-Fluorouracil, Dexamethasone) that inhibited the upregulation of these two miRNAs were identified, and Diethylstilbestrol, Etoposide, 5-Fluorouracil and Dexamethasone among them were confirmed their potential association with lung cancer based on some studies (45–48). Therefore, we believe that 17beta-estradiol (E2) may also have some potential efficacy on primary LUSC.

In the metastatic process of LUSC, “endothelial cell migration” and “cell adhesion” were regulated by lncRNA *RP11-286B14.1* which were up-regulated in the primary cancer, and were reversely down-regulated in the metastatic process (**Figure 6I**). It competed with mRNA *PRKX* and *ITGB8* for *miR-27a-3p*, *miR-27b-3p* (**Figure 6J**), and inhibiting the expression of the two miRNAs that would upregulate the expression of lncRNA *RP11-286B14.1*. Likewise, we found 17beta-estradiol (E2), 5-Fluorouracil, Dexamethasone, Vinblastine, 5-aza-2’-deoxycytidine (5-Aza-CdR), Tamoxifen, Budesonide, ACTH, Doxorubicin and Paclitaxel could inhibit upregulation of *miR-27a-3p* and *miR-27b-3p*. In addition to 17beta-estradiol (E2), all of those drugs were reported the correlation with lung cancer (47–55), suggesting that 17beta-estradiol (E2) might be used as a potential drug for metastatic LUSC treatment.

## Discussion

Lung cancer is one of the most common malignant tumors in the world currently. Lung cancer metastasis is the main cause of death in lung cancer patients. Therefore, it is crucial to study lung cancer carcinogenesis and metastasis. In our study, LUAD and LUSC datasets, which account for 85% of lung cancer, were used to investigate critical roles of lncRNAs in lung cancer carcinogenesis and metastasis. Importantly, there are several achievements in our study. First, we identified repeatedly dysregulated lncRNAs, indicating that these lncRNAs not only played a crucial role in carcinogenesis, but also played a critical role in cancer metastasis. Next, using ceRNA regulatory interactions, we analyzed the effects of regulatory changes in the regulation of lung cancer progression by repeatedly dysregulated lncRNAs during normal-primary-metastatic progress. In addition, we performed drug resetting and then identified candidate drugs that were associated with primary and metastatic lung cancer (LUAD, LUSC) through the predicted lncRNA-related ceRNA groups.

For 12 candidate lncRNAs of LUAD identified in our study, lncRNA *HOXA11-AS* has been shown an effect on NSCLC. LncRNA *LINC01013* enhanced invasion of human anaplastic large-cell lymphoma, while *RNF144A-AS1* and *LINC01013* were also highly expressed in ALCL, indicating the potential in ALCL migration (56); LncRNA *CEBPA-AS1* was found that could effectively predict prognosis of LUAD (57); Wang et al. provided evidence that angiogenesis in HCC is hindered by silencing of lncRNA *BZRAP1-AS1 (* 58); *LINC00843* was validated that could be used as novel epigenetic markers for gastric cancer (59). Furthermore, the majority of mRNAs involving in the primary or metastatic functional regulatory gain network had been shown the association with cancer, such as *TP53INP1* among the 14 mRNAs involving in the metastatic functional network had been shown in various studies concerning hepatocellular carcinoma and colorectal cancer (60, 61). In our functional regulatory network, *TP53INP1* was regulated by lncRNA *BZRAP1-AS1*, which affected metastasis-related autophagy. The mRNA *TP53* involving in the primary functional network regulated “cell cycle” and “proliferation” functions in the network and was regulated by lncRNA *CEBPA-AS1*. These studies and results indicated that these lncRNAs might play a key role in the process of LUAD metastasis.

Moreover, we found that *AC01847.3* among the 12 candidate lncRNAs was identified as a candidate biomarker in LUAD and LUSC, implying that though molecular expression and mechanism might differ greatly in LUAD and LUSC, some commonality presented. Besides, in LUAD, 17beta-estradiol (E2) was determined a potential candidate drug for both primary and metastatic LUAD treatment based on drug resetting of lncRNA *HOXA11-AS* and *RP11-408O19.5*. Notably, aiming at lncRNA *AC018647.3* and *RP11-286B14.1*, 17beta-estradiol (E2) was also predicted an impact on primary and metastatic LUSC by predictive drug resetting. These results completely confirmed therapeutic potential of 17beta-estradiol (E2) in lung cancer.

Recently, some researchers have pointed out that non-coding RNA can indeed be used as a drug target for disease treatment (62). This study found that the dysregulation of some lncRNAs that play important regulatory roles often leads to functional changes leading to cancer development or metastasis. If the drug can affect disease function by regulating the expression of the relevant lncRNA, the drug may be used as a candidate drug to treat the disease. Currently, there are limited studies focusing on targeted drugs related to lncRNA, and only a very small number of lncRNAs have targeted drugs. Consequently, we chose miRNAs regulating expression of lncRNAs as drug targets for drug resetting and screened for candidate targeted drugs having an impact on the competitive groups. Nevertheless, with the increase in research on lncRNA targeted drugs, it will be of major interests to develop drug resetting targeting lncRNA.

In conclusion, our study identified repeatedly dysregulated lncRNAs during lung cancer metastasis as well as carcinogenesis and potential therapeutic drugs that target these lncRNAs, and provided a reference for the study and clinical treatment of lung cancer pathogenesis. We reversed the dysregulation of lncRNA by drugs, thereby regulated the dysregulated pathways in cancer cells, and ultimately achieved the goal of disease treatment. This work can provide a new idea for the subsequent screening of lung cancer treatment drugs.

## Data Availability Statement

The original contributions presented in the study are included in the article/**Supplementary Material**. Further inquiries can be directed to the corresponding author.

## Author Contributions

YX conceived and designed the project. SD and CW conceived and designed the experiments and wrote the manuscript. SD and CW analyzed the data. LL, CS, and BQ revised the manuscript. All authors contributed to the article and approved the submitted version.

## Funding

This work was supported by the National Natural Science Foundation of China (grant numbers 81673036).

## Conflict of Interest

The authors declare that the research was conducted in the absence of any commercial or financial relationships that could be construed as a potential conflict of interest.
