# Supplementary material for: Identification of Primary and Metastatic Lung Cancer-Related lncRNAs and Potential Targeted Drugs Based on ceRNA Network
Source: Front Oncol. 2021 Feb 3;10:628930. doi: 10.3389/fonc.2020.628930 (PMC7886985; doi:10.3389/fonc.2020.628930)
Supplement: Supplementary file 1 [file DataSheet_1.docx]

**Supplementary Material**

**Supplementary Table 1: Clinical information of lung cancer samples.**

| **Clinical Information(TNM, Stage)** | **LUAD** | **LUSC** |
| --- | --- | --- |
| Stage1 (T1,2N0M0) | 177 | 188 |
| Stage1 (T1,2NxMx) | 91 | 47 |
| Stage1 (N1) | 2 | 1 |
| Stage2 (N1,2) | 79 | 96 |
| Stage2 (T2,3NxMx) | 17 | 12 |
| Stage2 (T2-3N0M0) | 26 | 45 |
| Stage3/Stage4(T4/N1,2,3M1) | 108 | 88 |
| Stage3/Stage4(T2,3/N0M0) | 0 | 5 |
| No Stage | 2 | 3 |
| Summary | 502 | 485 |
| Primary | 177 | 188 |
| Metastasis | 187 | 184 |

**Supplementary Table 2: The survival comparison between primary and metastatic samples.**

| **Cancer** | **Stage** | **sample** | **3 year dead Ratio (%)** | **Mean dead day (Month)** |
| --- | --- | --- | --- | --- |
| LUAD | Primary | 168 | 10.12 | 41.81 |
|  | Metastasis | 175 | 32.57 | 22.94 |
| LUSC | Primary | 102 | 25.49 | 33.03 |
|  | Metastasis | 82 | 34.14 | 23.66 |

**Supplementary Table 3: Samples’ information and the number of mRNAs and lncRNAs in ceRNA networks.**

| **Cancer** | **Status** | **Sample** | **Competitive pairs (lncRNA-mRNA)** | **lncRNA** | **mRNA** |
| --- | --- | --- | --- | --- | --- |
| LUAD | Normal | 58 | 22,246 | 626 | 2,788 |
|  | Primary | 168 | 11,605 | 689 | 2,444 |
|  | Metastasis | 175 | 14,744 | 770 | 3,055 |
| LUSC | Normal | 17 | 4,887 | 581 | 2,086 |
|  | Primary | 102 | 12,030 | 789 | 2,813 |
|  | Metastasis | 82 | 8,872 | 709 | 2,538 |

**Supplementary Table 4: Differentially up/down-regulated lncRNAs in lung cancer between different statuses.**

| **Cancer** | **Normal/Primary/Metastasis** | **LncRNA(up/down)** | |
| --- | --- | --- | --- |
| LUAD | Normal VS Primary | 1867↑ | 1215↓ |
|  | Primary VS Metastasis | 128↑ | 301↓ |
| LUSC | Normal VS Primary | 2007↑ | 1273↓ |
|  | Primary VS Metastasis | 53↑ | 169↓ |

**Supplementary Table 5: The number of edges, lncRNAs, and mRNAs involved in the networks.**

| **Cancer** | **Normal/Primary/Metastasis** | **Gain/loss** | **Edge** | **lncRNA** | **mRNA** |
| --- | --- | --- | --- | --- | --- |
| LUAD | Normal VS Primary | gain | 305 | 33 | 261 |
|  |  | loss | 256 | 17 | 236 |
|  | Primary VS Metastasis | gain | 169 | 29 | 159 |
|  |  | loss | 197 | 29 | 177 |
| LUSC | Normal VS Primary | gain | 82 | 16 | 81 |
|  |  | loss | 54 | 12 | 54 |
|  | Primary VS Metastasis | gain | 35 | 12 | 35 |
|  |  | loss | 72 | 16 | 71 |
